# Development of a Non-Contact Flow Sensor Based on a Permanent Magnet Metal Clip for Monitoring Circulation Status

**DOI:** 10.3390/bios16020078

**Published:** 2026-01-27

**Authors:** Kicheol Yoon, Seung Hee Choi, Tae-Hyeon Lee, Sangyun Lee, Sunghoon Kang, Sun Jin Sym, Kwang Gi Kim

**Affiliations:** 1 Gachon Biomedical Convergence Institute, Gachon University Gil Medical Center, Incheon 21565, Republic of Korea; kcyoon98@gilhospital.com; 2 Medical Devices R&D Center, Gachon University Gil Medical Center, 21, 774 beon-gil, Namdong-daero, Namdong-gu, Incheon 21565, Republic of Korea; seankg2000@gmail.com; 3 Department of Electronic Engineering, Gyeonggi University of Science and Technology, 269 Gyeonggigwagi-dearo, Siheung City 15073, Gyeonggi-do, Republic of Korea; shchoi@gtec.ac.kr (S.H.C.); thlee@gtec.ac.kr (T.-H.L.); 4 Division of Medical Oncology, Department of Internal Medicine, Gachon University Gil Medical Center, 21, 774 beon-gil, Namdong-daero, Namdong-gu, Incheon 21565, Republic of Korea; 5 Department of Radiological Science, Dongnam Health University, 50 Cheoncheon-ro 74 gil, Jangan-gu, Suwon 16328, Gyeonggi-do, Republic of Korea; leesy2024@dongnam.ac.kr; 6 Department of Biomedical Engineering, Gachon University, 1342 Seongnam-daero, Sujeong-gu, Seongnam-si 13120, Gyeonggi-do, Republic of Korea; 7 Department of Health Sciences and Technology, Gachon Advanced Institute for Health Sciences and Technology (GAIHST), Gachon University, 38-13, 3 Dokjom-ro, Namdong-gu, Incheon 21565, Republic of Korea

**Keywords:** multiple punctures, non-contact flow sensor, permanent magnet, mu-metal, Lorentz force

## Abstract

Foreign matter accumulating on catheters during ascites paracentesis in cancer patients can interfere with the procedure. The paracentesis site must be visually inspected by patients or medical staff. We propose a monitoring method using sensors, as they enable real-time, automatic status detection. The proposed design integrates a sensor into the drainage tube to detect liquid flow using the Lorentz force. The sensor consists of a permanent magnet, a yoke, and a signal processing circuit. Mu-metal shields the magnet to prevent interference with surrounding circuits. Physiological saline solution is used as a substitute for bodily fluids. Sensor performance was evaluated via finite element analysis. The Lorentz force generated an average voltage of 11.07 μV when liquid was present, enabling detection of the flow status. The proposed sensor is non-invasive and features a clip design, allowing attachment and detachment from the drainage tube for reuse. Non-invasiveness ensures safety from infection, and reusability can reduce medical costs. This study proposes a sensor for monitoring peritoneal puncture status. By detecting liquid flow using the Lorentz force, the system enables real-time monitoring during the procedure.

## 1. Introduction

Malignancy-related ascites (MA) accounts for approximately 10% of all ascites cases, in patients with ovarian adenocarcinoma, followed by breast cancer, colon cancer, gastric cancer, and pancreatic cancer [1]. MA reduces quality of life due to abdominal distension (55%), abdominal pain (53%), nausea (37%), anorexia (36%), vomiting (25%), fatigue (17%), dyspnea (11%), early satiety (6%), and weight changes (5%) [1].

The most common treatment for malignancy-related ascites is intermittent paracentesis, where even small-volume drainage (>2.0 L) can relieve abdominal distension in terminal cancer patients (>90%) [1,2,3]. Ascites can be drained by directly inserting a catheter into the abdominal cavity and connecting a drainage tube and collection bag [2]. However, repeated procedures are often inevitable [4]. During paracentesis, problems may arise due to pus, blood clots, stones, tissue fragments, or debris, which can easily obstruct the drainage tube. Kinking of the tube may also cause obstruction, making drainage difficult. Therefore, tube blockage can lead to paracentesis failure, local edema, and infection, potentially threatening the patient’s life if not properly managed [5]. Although medical staff can check the tube periodically and patients can visually inspect the tube and notify staff, this places a burden on healthcare providers and may limit patient comfort [5]. Additionally, patients who are asleep or have impaired levels of consciousness may not be able to monitor themselves, further increasing the workload for medical personnel and limiting the quality of care [5]. Consequently, there is a need to develop a system capable of the continuous real-time monitoring of drainage tubes to alert medical staff during paracentesis and allow timely intervention in case of blockage. While research and development on the monitoring of intravenous (IV) infusion exist, systems for the real-time monitoring of ascites drainage are still lacking [6,7,8,9,10]. Pressure sensors have been applied to chest drainage systems to detect real-time pleural pressure changes, assisting medical staff in assessing patient status. Similarly, sensors have been developed to monitor cerebrospinal fluid drainage and intracranial pressure [11,12]. However, the development of real-time monitoring sensors for ascites drainage remains insufficient. Some studies have explored capacitive sensor sleeves for continuous intra-abdominal pressure (IAP) monitoring, smart sensors for real-time postoperative abdominal drainage monitoring, capacitance-based methods for detecting drainage tube flow, and ultrasound-based real-time monitoring to address catheter obstruction during paracentesis [13,14,15,16,17,18].

Capacitive sensor sleeves are thin, flexible, and non-invasive [13]. They can continuously monitor in real time without interfering with catheter function and can be directly integrated with internal catheters [13]. Capacitance-based methods using interdigital electrodes allow real-time monitoring of drainage status [14]. Implantable sensors, although invasive, can be inserted into the abdominal cavity and linked to an external drive using cables, enabling paracentesis at any time and place [15]. Smart sensors, using sensors and spectrometers, allow real-time measurements at the patient’s bedside and enable objective digital management [16]. Ultrasound-based real-time monitoring utilizes point-of-care ultrasound (POCUS) to address catheter obstruction [17].

Invasive sensors involve high technical complexity [13,14]. Challenges include evaluating patient-specific immune reactions to materials, electrical impedance changes due to protein adsorption, infection risks, corrosion due to abnormal pH, and potential inflammatory or allergic responses [19,20,21,22]. In narrow clinical or surgical spaces, cables may pose tripping hazards, disrupt procedures due to signal disconnection, and increase infection risk through direct contact with the floor [23,24]. Repeated connector usage can damage connectors or insulation, degrade signal performance, cause malfunction, and generate noise, interfering with procedures [25].

An interdigital capacitor for invasive sensors can be fabricated as a cylinder to allow miniaturization and integration [14], enabling a high-quality factor (Q) capacitance effect without parasitic components, which is expected to yield high sensing performance [26,27]. However, dielectric constant variation due to temperature, dielectric loss (especially heat sensitivity), and increased interdigital finger count may enlarge the sensor, potentially interfering with its invasive purpose [27,28,29]. POCUS monitoring requires the presence of medical staff, which can be inconvenient, labor-intensive, and space-consuming [17]. Smart drain sensors integrate the sensor with the drainage tube, allowing detachable drives using magnets. While the drive is reusable, the sensor must be discarded with the tube due to infection concerns [18]. Therefore, developing a small, lightweight sensor and monitoring system that is detachable, attachable, reusable, and safe from infection is crucial.

This paper proposes a clip-type sensor and liquid flow detection method based on a compact, lightweight design that allows for detachment and reattachment of the drainage tube, is safe from infection, and is reusable. The proposed method aims to investigate the feasibility of detection by applying finite element analysis based on the Lorentz force principle of the liquid flow state sensed by the proposed sensor.

## 2. Principle-Based Analysis of Liquid Flow Detection Method

**Figure 1:**
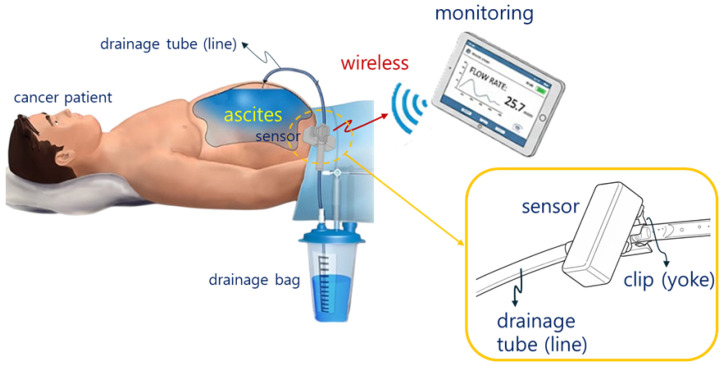
Wireless monitoring concept for multiple patient or device states.

The current multiple-site aspiration procedure involves draining 2 L over 2 h, as shown in Figure 1. Even during a seemingly smooth drainage process, situations can occur where drainage suddenly stops.

**Figure 2:**
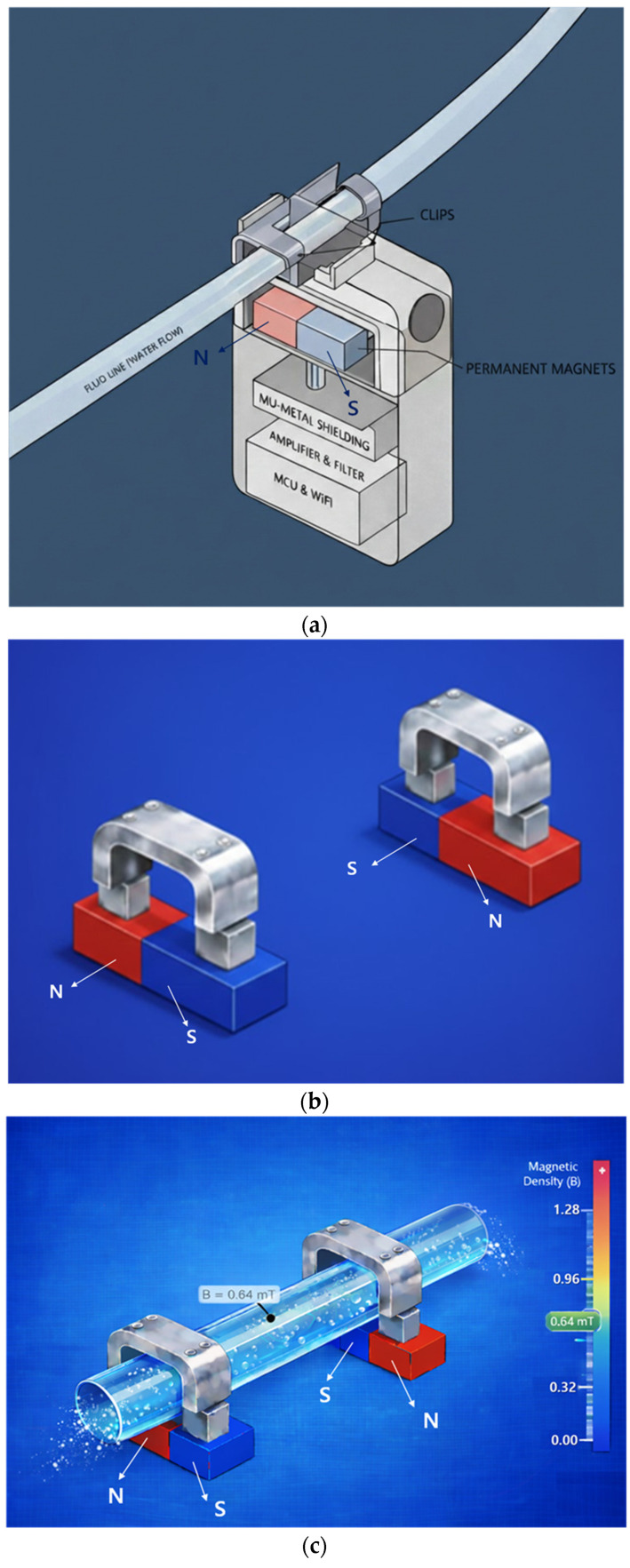

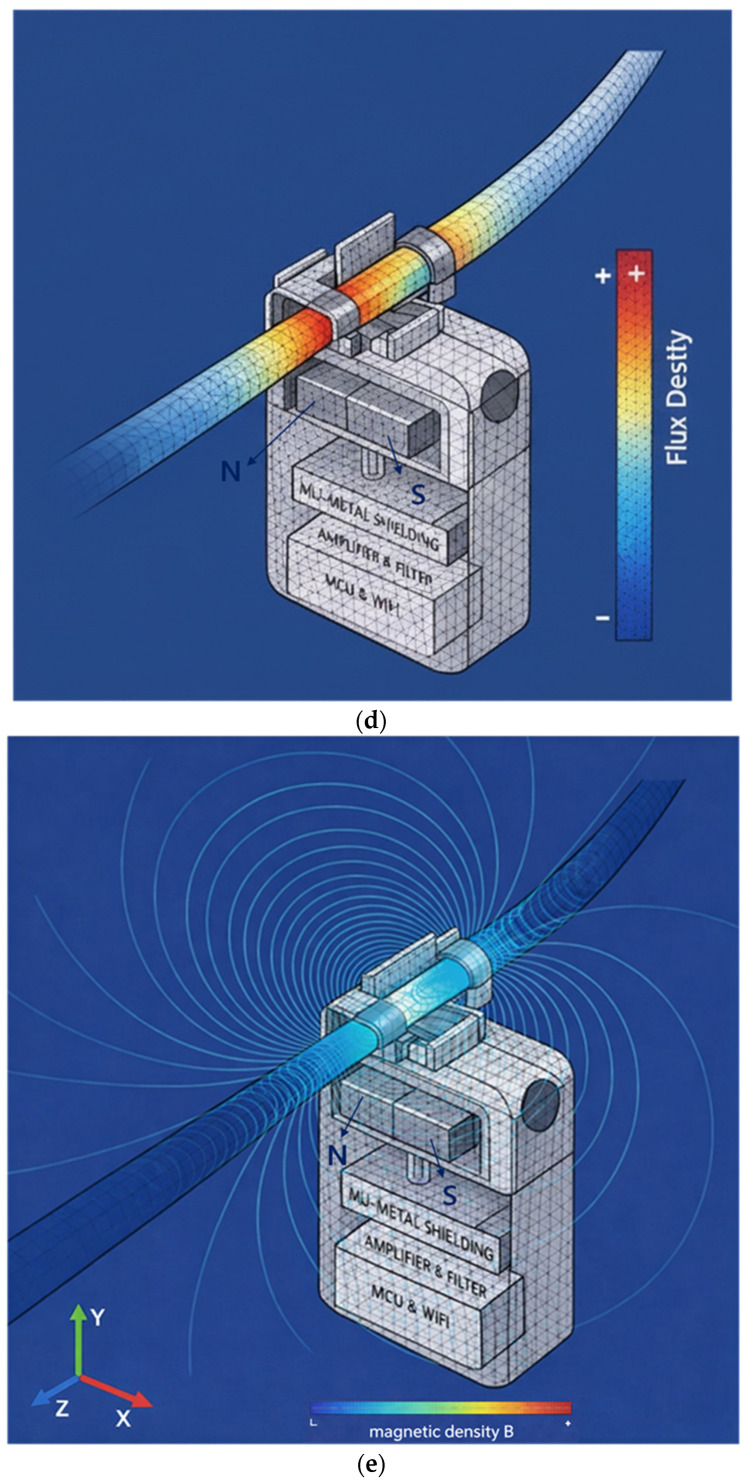

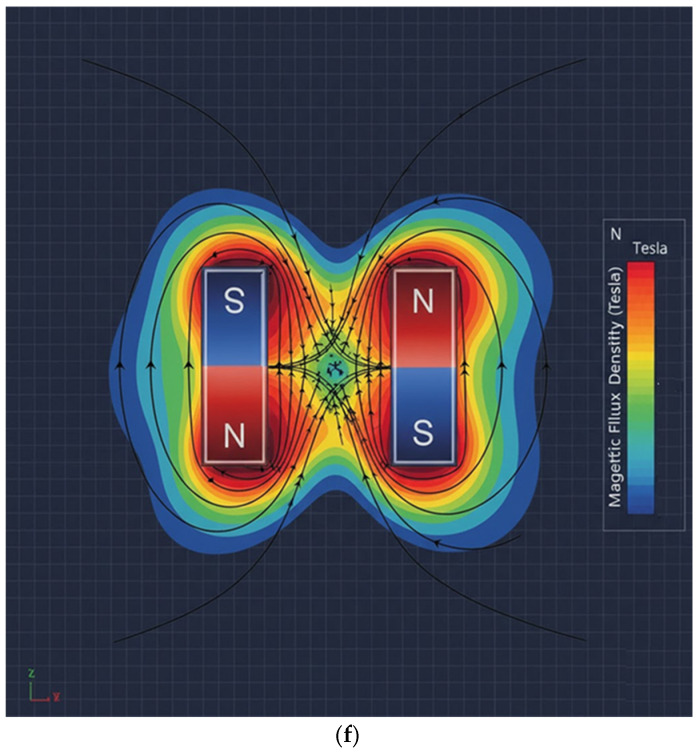
Proposed sensor structure (magnet dimensions: width 20 mm, height 0.5 mm, thickness 0.25 mm): (**a**) overall schematic of the sensor device, (**b**) connection between magnet and yoke, (**c**) drainage tube connected to yoke and magnet structure, (**d**) FEA of flow rate in the drainage tube, (**e**) FEA of magnetic field distribution, and (**f**) FEA illustrating magnet operating principle.

The sensor consists of a permanent magnet, a metal clip (CLP) that functions as mu-metal and a magnetic yoke, a Lorentz force sensing electrode, a signal control and amplification circuit, and a Wi-Fi module for wireless communication, as shown in Figure 2a.

The permanent magnets are connected using two permanent magnets and two yokes (clips) as shown in Figure 2b, and the permanent magnets are arranged facing each other. It is important to note that although the permanent magnets face each other, they are arranged with their N- and S-poles facing each other, which is fundamental to the magnets’ performance. In Figure 2a, the permanent magnets face each other with a fixed gap behind them. Since the external surface appears to them as a single magnet, the detailed structure shown in Figure 2b is presented. Here, the yoke is configured as a clip, allowing a drainage tube to be attached, as shown in Figure 2c, enabling liquid to flow through the yoke. The drain tube is primarily composed of insulating material.

In the magnetic field path, if liquid flows through the drain tube as shown in Figure 2d, the magnetic field propagates from the N-pole of the permanent magnet, forming a closed magnetic circuit that returns to the S-pole as shown in Figure 2e. That is, when two magnets exist as shown in Figure 2f, the magnetic field propagates to the adjacent magnet, forming a closed loop.

**Figure 3:**
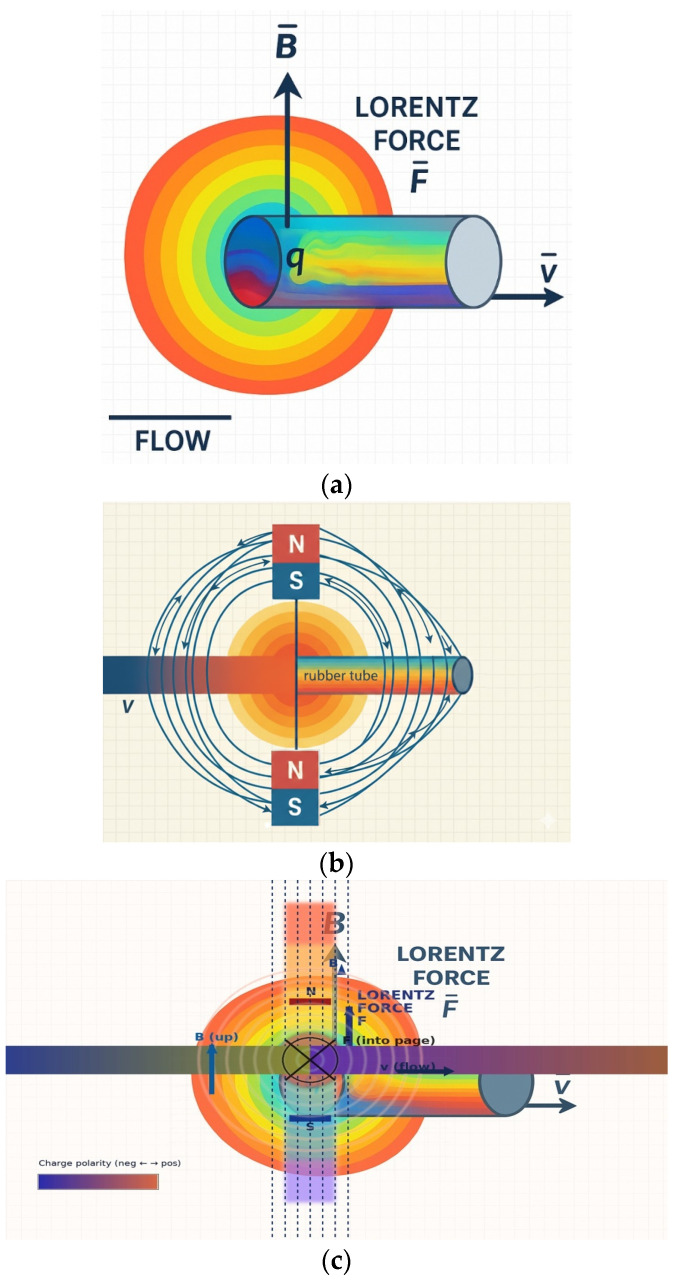
Magnetic field generation and Lorentz force-based liquid flow detection in the proposed sensor structure: (**a**) Lorentz force phenomena in flowing liquid, (**b**) magnetic field generated by the sensor magnet, and (**c**) orthogonal relationship between the magnetic field and Lorentz force.

Mu-metal (M_u_) is applied to effectively shield the leakage flux of magnets, forming a concentrated closed path. Since mu-metal is inserted between the magnet and surrounding components, it effectively blocks leakage flux. Referring to Figure 2c,d, the metal clip functions as a yoke (width: 10.8 mm, height: 0.3 mm, thickness: 0.12 mm) to mechanically secure the drainage tube (diameter: 4 mm). Therefore, the magnetic field generated in this structure, as shown in Figure 2e, forms along the direction of the drain tube (X-axis) in the yoke, which is the detection region where the liquid flows, and exhibits a circular distribution in the cross-sectional plane of the drain tube (Y–Z plane). Penetration of the magnetic field into the system is significantly suppressed because the magnetic flux lines are diverted to follow the interior of the μ-metal shielding structure positioned on the system side (Y-axis). Consequently, the magnetic field is not radiated as a directional beam but is instead induced and concentrated along the direction of the drain tube sensing area (X-axis) under shielding conditions. The clip is designed with a structure that surrounds the tube. The liquid (L_q_) flowing inside the drainage tube carries a charge (q = 4.82 × 10^−4^ C) containing ions, as shown in Figure 3a. The flow rate was measured using an ultrasonic flow sensor (FDH-22F, KOREA KEYENCE Co., Ltd., Seonnam, Republic of Korea). The measured average flow rate (Q) is 2.78 × 10^−7^ m^3^/s (Q = 2.78 × 10^−7^ m^3^/s). At this time, the flow velocity (V_q_) is analyzed to move in the direction (x-axis) at 0.088 m/s (V_q_ = 0.088 m/s) as shown in Equation (1) [30]. Therefore, when the charge passes through the magnetic field (B_T,y_) at a 90° orthogonal angle, the Lorentz force (F = 2.12 μN) is analyzed to occur as shown in Equation (2) [31].


(1) $$V_{q}=\frac{Q}{A}=\frac{4Q}{\pi D^{2}}\left[m/s]\;@\;A_{TB}=\frac{\pi D^{2}}{4}[m^{2}\right]$$

The charge (q) interacts with the magnetic field (B) formed by the magnet, as shown in Figure 3b, and experiences the Lorentz force (F). This force acts in a direction perpendicular to both the flow velocity (v) and the magnetic field (B) [31].

Here, the Lorentz force (F) acts in a direction perpendicular to both the flow velocity (V_q_) and B_T_ (z-axis), as shown in Figure 3c and expressed by Equation (2). This force separates the positive ions (+q) and negative ions (−q) within the liquid and accumulates them toward the clip electrodes, thereby separating the charges. This separation and accumulation of charge creates an induced potential difference (V_Lq_) between the two clip electrodes, as shown in Equation (3). This potential difference (V_Lq_) is proportional to the flow velocity (V_Lq_) and serves as the primary detection signal for analyzing the flow rate (Q) [32]. Here, L_ind_ is the effective distance between the electrodes.


(2) $$F=q(V_{q}\times B_{T})\;@\;B_{T}=0.64\;mT$$

(3) $$V_{Lq}=V_{q}B_{T}L_{ind}$$

These sensors feature highly complex structures, and due to interference from surrounding circuits, theoretical analysis regarding the shielding effect of mu-metal and the performance of the yoke can serve only as a reference. Mathematical and physical theoretical analysis has limitations in describing the actual phenomena occurring. Therefore, it is necessary to perform simulations and obtain results through finite element analysis to investigate the correlation between liquid flow and the Lorentz force.

## 3. Finite Element Analysis and Measurement Results of Liquid Flow Detection

**Figure 4:**
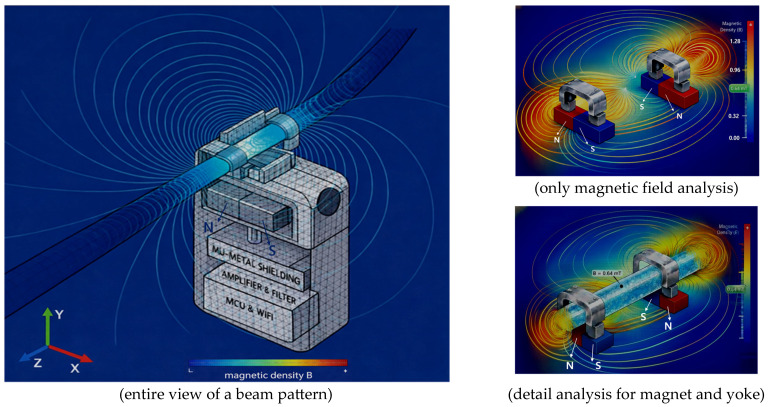
Simulation of magnetic field distribution and Lorentz force in liquid flow around the sensor using finite element analysis.

The sensor structure includes a non-uniform air gap called the drain tube (T_B_), making analysis of the magnetic field distribution complex. Therefore, this structure is analyzed to evaluate the spatial distribution of magnetic flux density (B_T_) in detail by performing finite element analysis (FEA) on the charge and magnetic field distribution phenomena, as shown in Figure 4, with the magnet, yoke, and drain tube installed inside the system shown in Figure 1.

The FEA results clearly show that magnetic flux is effectively concentrated inside the flow channel (T_B_) compared to the external clip area. This analysis preliminarily verifies the potential weakening of the internal magnetic field due to the minute air gap in the external clip and identifies the stable magnetic field formation required for flow detection.

The sensor exhibited FEA simulation results, demonstrating that a uniform and stable magnetic flux density (B_T_) of approximately 0.64 mT is formed within the flow detection zone (interest zone) inside the tube.

**Figure 5:**
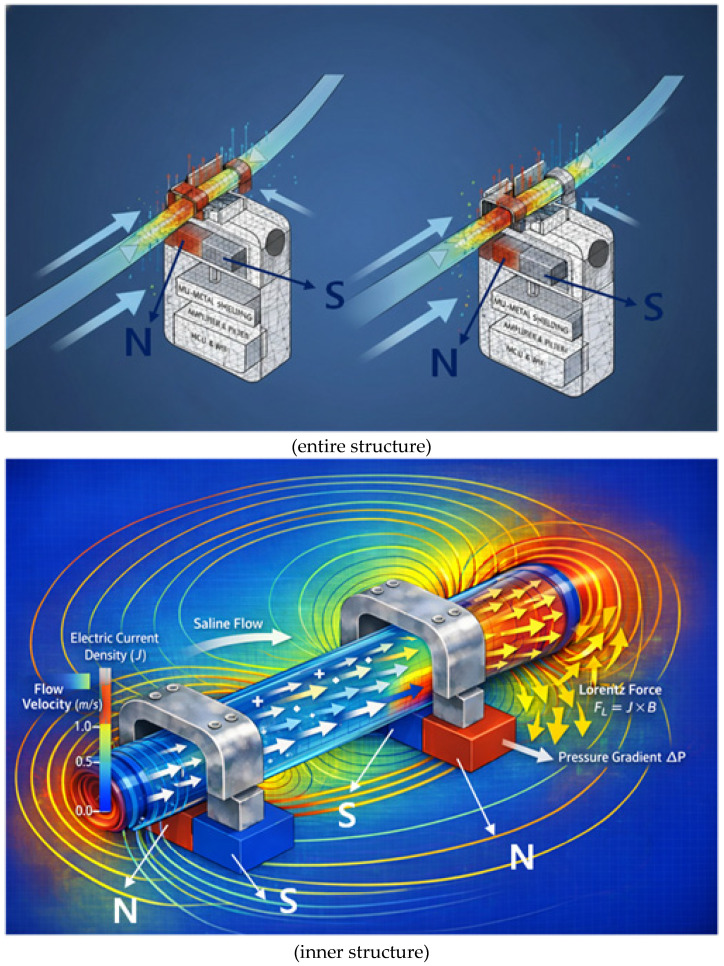
Qualitative visualization of magnetohydrodynamic flow from finite element analysis, showing the Lorentz force (J × B) acting on the conductive fluid and driving pressure-driven flow.

This sensor utilizes a flow detection principle based on the Lorentz force, as shown in Figure 5, and thus it was necessary to emphasize the need for FEA based on flow rate changes (V_Lq_). The Lorentz force was implemented in the fluid momentum equation as a volumetric body force term f = J × B, where the electric current density J and magnetic flux density B were obtained from the coupled electromagnetic finite element solution. The visualization shown in Figure 5 represents a qualitative rendering of these numerically computed fields rather than a direct element-wise contour plot.

To facilitate interpretation, we have endeavored to enhance understanding by presenting both a two-dimensional cross-section (single-sided analysis) and a two-sided (double-sided analysis). We aim to analyze in detail the principle of the Lorentz force (F) generated when a conductive liquid flows at a velocity (v) within a magnetic field (B). This principle utilizes the Lorentz force induced when a fluid flows within a magnetic field formed by a permanent magnet to convert the fluid flow state into a non-contact voltage signal. Charges within the moving liquid are deflected by the Lorentz force in a direction perpendicular to both the flow direction and the magnetic field direction. This creates a voltage difference across the tube ends. Since the magnitude of the generated voltage is proportional to the flow velocity (v), precise measurement of this voltage allows real-time calculation of the drainage status and flow rate.

**Figure 6:**
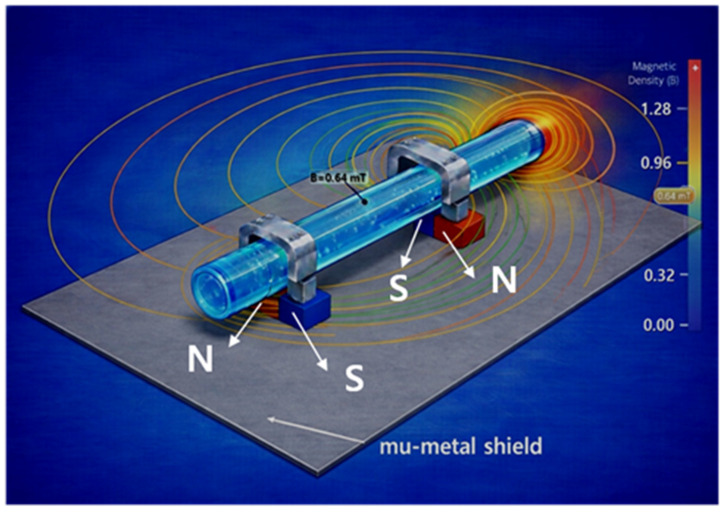
Simulation of magnetic field distribution with mu-metal shielding planes.

This is achieved by using a metal yoke to concentrate the magnetic flux within the tube, enabling the detection of even minute changes in drainage flow rate through variations in the Lorentz force. Furthermore, as shown in Figure 6, the sensor exhibits a beam pattern characteristic where the magnetic field propagation pattern is concentrated in the direction of the flow channel due to its U-shaped mu-metal shield structure.

Therefore, it has the advantage of enhancing sensitivity by maximizing the magnetic field strength within the flow channel while simultaneously reducing noise by shielding the surrounding circuitry from external magnetic field interference. That is, because the sensor is surrounded by mu-metal, the shielding protects the surrounding circuitry from interference.

This Lorentz force separates and accumulates positive and negative ions within the fluid toward the electrode direction, thereby generating a Lorentz-induced voltage (V_Lq_ = 11.05 μV) proportional to the flow velocity.

(1) Primary detection signal: flow analysis based on induced potential difference (V_Lq_)

**Figure 7:**
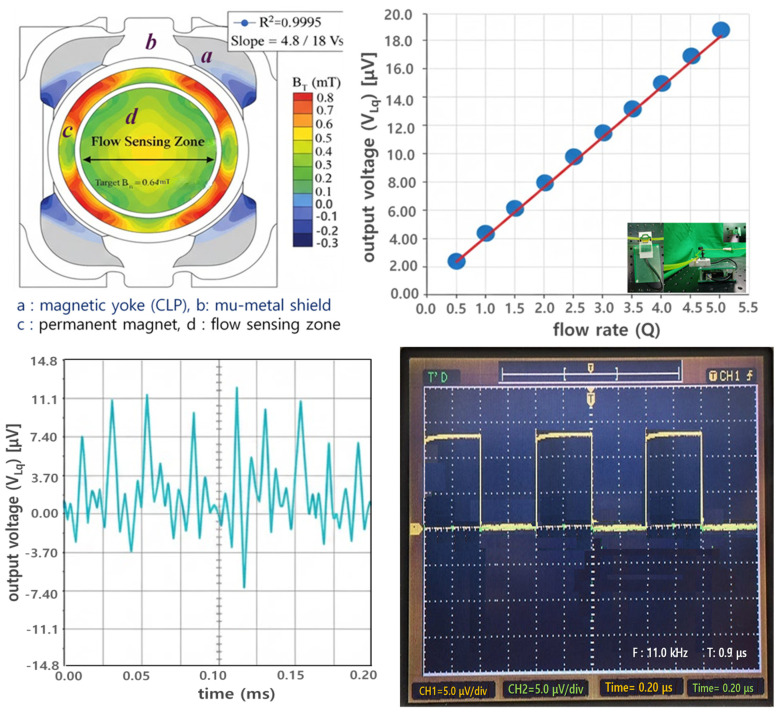
FEA and voltage simulation results of the magnetic flux density (B_T_) in the flow sensor, showing flow linearity and time-response characteristics [Video S1].

We aim to determine the magnitude of the primary detection signal through FEA results. With B_T_ set to 0.64 mT and flow velocity (V_q_) at 0.088 m/s (flow rate Q = 2.78 × 10^−7^ m^3^/s), the induced voltage difference (V_Lq_) is calculated as 11.05 μV. Such a minute signal can be amplified to the mV (or V) level via an amplification circuit. Figure 7 shows the magnetic flux density distribution across the flow channel cross-section calculated via finite element analysis (FEA). The mu-metal shielding structure concentrated the magnetic flux density within the flow sensing area uniformly and stably at approximately 0.64 mT. This demonstrates the establishment of a constant magnetic field environment suitable for Lorentz force-based flow detection.

The output voltage measured from the figure according to the Lorentz force principle exhibited very high linearity with flow rate changes, allowing us to assess the sensor’s voltage response characteristics to flow rate.

Through mathematical analysis, the results indicate that when flow rate (Q), flow velocity (V_q_), and magnetic flux density (B_T_) are 2.78 × 10^−7^ m^3^/s, 0.088 m/s, and 0.64 mT, respectively, the induced voltage (V_Lq_) is 11.05 μV under flow conditions and approaches 0 μV under no-flow conditions. However, when obtaining simulation results, errors may occur due to the influence of the surrounding environment (including air, module interference effects, coupling loss, radiation loss, conduction loss, material intrinsic resistance loss, material dielectric loss, and fringing field [33]), potentially yielding values slightly lower than the theoretical values, as shown in Table 1. In the case of measurement results, errors due to coupling loss and impedance mismatch between the connector and the module can cause the results to be even lower than the simulation results. Therefore, it is necessary to analyze the results considering this error rate. It is important to note that in actual no-flow conditions, both simulation and measurement results cannot reach 0 μV but can only approach 0 μV. Consequently, the error rate for the results must be defined as within 5% to ensure normal operation without issues.

(2) Verification of sensor performance centered on Lorentz force voltage signals based on magnetic fields

Ionic liquids such as physiological saline have a very high relative permittivity (ε_r_ ≈ 80), and the measured capacitance values are largely determined by the dielectric properties of the liquid itself, as shown in Table 2 [34,35]. Converted to absolute permittivity, ε_Lq_ = ε_r_ε_0_ ≈ 7.08 × 10^−10^ F/m, indicating that the drainage tube material itself (e.g., PVC) has negligible influence on the capacitance. Therefore, while capacitance changes are useful for detecting liquid presence, they provide limited accuracy for determining dynamic information such as flow rate or velocity.

**Figure 8:**
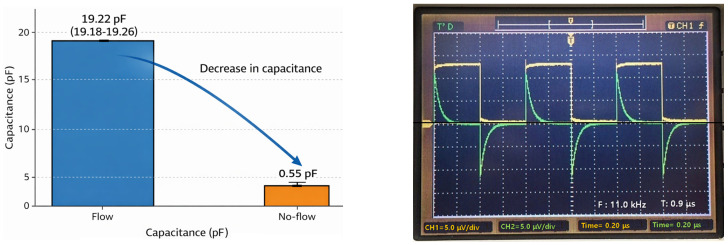
Capacitance measurement for detecting liquid presence in a drainage tube, used for comparison with the sensor system.

The C_Lq_ values when the drain tube was filled with liquid and when it was filled with air were experimentally and computationally determined as shown in Figure 8. In the presence of liquid, C_Lq_ ≈ 19.1 pF, while when filled with air, C_Lq_ was measured to be approximately 0.2–0.8 pF, approaching zero.

This value was used to compare the capacitance with the reference/comparison indicator by comparing it to the Lorentz force voltage signal obtained from the sensor, as shown in Table 3, and was not used for actual liquid flow detection. The ±5% level of error that occurred during the simulation and measurement process can be considered acceptable.

Actual sensor performance verification is primarily conducted based on the voltage signal generated by the Lorentz force acting on moving ion charges in a magnetic field. This voltage signal is directly linked to the fluid flow velocity and flow rate; it is zero when no liquid is present. Therefore, the proposed magnetic field-based sensor can simultaneously detect not only the presence or absence of liquid but also flow rate and flow velocity information.

Capacitance-based sensing methods utilize the dielectric constant difference between air and liquid to detect the presence of liquid inside a tube. They have been widely used primarily in liquid level sensors or for detecting the presence of fluid [36,37]. Due to the significant dielectric constant difference between air and liquid, this method is effective for distinguishing between an empty drainage tube and one filled with liquid [36].

## 4. Discussion

This study presents a method for detecting liquid flow using sensors. For the experiment, actual ascites fluid was not used; instead, physiological saline solution was employed as a substitute. Should patient ascites fluid be utilized in the future, it would require IRB review for use; therefore, the results will be addressed in future studies. In the experiment, physiological saline solution was continuously circulated via a catheter drainage tube (length 2 m, diameter 6 mm) using a motor to maintain a steady flow rate.

The liquid flows through a drainage tube, which utilizes PVC tubing lines commonly used in intravenous fluid administration [38]. This tubing line is attached to a clip mounted on the sensor. The method defines liquid flow detection as the Lorentz force (F), defined by the 90° orthogonal relationship between the magnetic field generated by a magnet and the charge movement due to the liquid flow.

**Figure 9:**
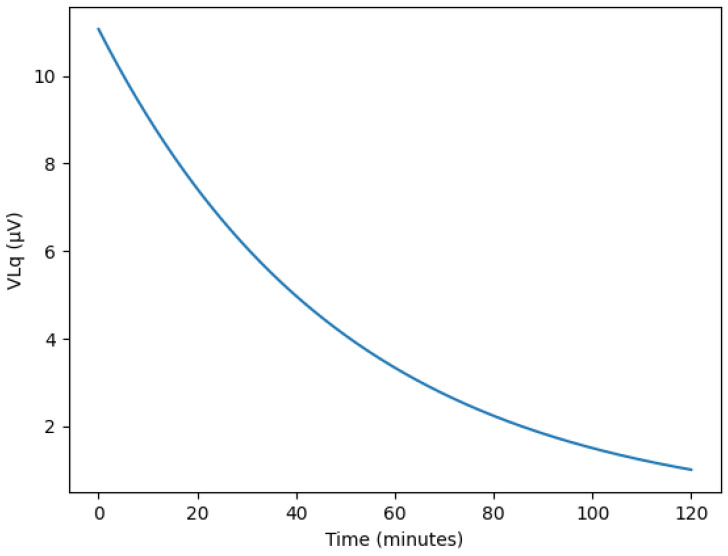
Simulated temporal variations in Lorentz-induced voltage (V_Lq_) during continuous ascites drainage, showing exponential decay under pressure-driven flow reduction.

During actual paracentesis, the flow rate exhibits an exponential decrease over time, as shown in Figure 9. Initially, the liquid flows rapidly due to the pressure difference between the body and the drainage tube. However, as the drainage bag gradually fills, the pressure difference decreases, causing both the flow rate and velocity to diminish progressively. Typical peritoneal dialysis drains approximately 2 L of fluid over about 2 h. During this process, as the drainage bag fills, the charge associated with the liquid flow decreases. Consequently, the induced voltage measured by the sensor, based on the Lorentz force, also changes over time.

The induced voltage calculated using the Lorentz force principle is extremely minute, at approximately 11.05 microvolts. An amplification circuit is essential to reliably detect these small signals and apply them to the monitoring system. In this study, the amplification circuit was designed to increase the voltage level from the millivolt range to the volt range, facilitating subsequent signal processing.

**Figure 10:**
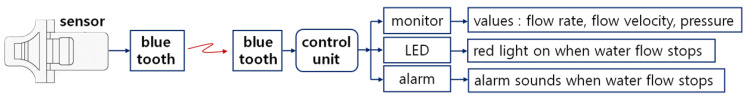
Schematic diagram of the wireless sensor system for monitoring liquid flow status [Video S1].

The sensor must be detachable and attachable to the drainage tube, and it must be small and lightweight. Furthermore, using a sensor that is easy to connect to the drainage tube, safe from patient infection, and reusable is highly convenient for medical procedures and enables the provision of optimal treatment services to patients. To design the monitoring system, the priority should be designing a small, lightweight sensor with a ring-shaped structure that allows for detachment and attachment to the drainage tube. The sensor can be technically expanded for application in the medical field, as illustrated in Figure 10 [Video S1]. For example, a systematic design sequence involves incorporating a wireless communication module and utilizing a control unit, warning lamp, warning sound, and display monitor. This setup displays the drainage status on the monitor when drainage is occurring and activates a normal lamp. If drainage is obstructed, the warning lamp illuminates and the warning sound activates, enabling both the patient and medical staff to recognize the issue. The LCD monitor can provide flow rate, velocity, and pressure information. When fluid flows smoothly, it displays flow rate, velocity, and pressure values. However, if the drainage tube becomes blocked and fluid flow stops, it displays values approaching zero for flow rate, velocity, and pressure. The LED consists of two types: a green LED illuminates when fluid flows smoothly, and a red LED illuminates when fluid flow ceases. At this time, the alarm can also be configured to emit a warning sound.

The need to develop a wireless communication-based monitoring system to verify the status of fluid drainage stems from several causes.

Healthcare providers often observe patients directly by staying by their side or visiting at set times, while patients frequently request assistance after personally noticing and witnessing issues. However, daily observation within healthcare providers’ busy schedules increases workload, and limitations arise in assessing the situation if patients fall asleep or experience decreased consciousness [5]. Furthermore, patients who are asleep or have impaired consciousness cannot be monitored easily, increasing the burden on medical staff and limiting the quality of care provided [5]. Therefore, developing a sensor-based wireless communication monitoring system that provides observation, detection, and alerts by monitoring the drainage tube in real-time to notify medical staff about the paracentesis process and enable appropriate treatment for drainage blockages would be highly effective. This method would also enable remote monitoring by medical staff and be highly efficient for checking drainage status while patients with impaired consciousness or sleeping patients remain in a stable state.

The differences between existing studies and this study are summarized and recorded in Table 4.

Refs. [6,7] used invasive methods based on pressure sensors, posing infection risks, while [8] required expensive equipment and complex installation processes. The smart drain system could not perform real-time remote monitoring due to its wired connection [9,10] used disposable sensors, resulting in poor cost-effectiveness. However, this study employs a non-invasive sensor attachable to the exterior of the drainage tube. It enables real-time monitoring of drainage status via Bluetooth-based wireless communication. Its reusable design minimizes infection risk while simultaneously securing economic advantages. Therefore, it significantly enhances patient safety and convenience while overcoming the limitations of existing methods.

## 5. Conclusions

The primary focus of this study is non-invasive technology. While invasive or implantable sensors can offer convenience in patients’ daily lives, they may cause discomfort requiring reoperation to replace the sensor, due to issues such as lead wire disconnection for component connection caused by pH values, intestinal movement, nerve or vascular adhesions, and serum; rapid battery depletion due to body heat and acidity; and toxicity and infection problems arising from corrosion of component materials [39,40].

This paper proposes a sensor design that can be detached and reattached to the drainage tube during the peritoneal puncture process.

The proposed sensor utilizes wireless communication to assemble modules such as a warning sound indicating drainage status abnormalities, an LCD monitor displaying drainage flow rate, velocity, and pressure, and alerts via a control board. This expands its application scope for monitoring peritoneal dialysis status.

Developing such a system is highly beneficial for patients and medical personnel. It enables safe monitoring of flow status when patients are unconscious or asleep, alerting medical staff to potential problems and facilitating timely intervention. The reference content of this experiment aims to evaluate detection performance for drainage status using physiological saline instead of fluid, and monitoring performance via wireless communication. This is because the primary reason for evaluating the liquid detection performance of the clip sensor is its non-contact detection through the drainage tube.

The sensor is designed as a clip type, allowing attachment and detachment to the drainage tube via a clip, enabling reuse. It is also considered excellent for infection prevention and hygiene management through ethanol spray disinfection. The design utilizes a hybrid module connection, enabling easy system design at a low cost. Furthermore, redesigning the sensor system as a chip form allows for on-device design or semiconductor chip fabrication. Integrating it onto a PCB board through this process is expected to increase mass production and distribution to medical institutions. This will enhance its applicability in various forms, such as multiple punctures, brain tumor treatment, IV therapy, and urine drainage.

## Figures and Tables

**Table 1:** Analysis of Lorentz-induced voltage (V_Lq_).

Detection Signal	Calculation	Simulation Results				Measurement Results			
		Min.	Avg.	Max.	Error Rate [%]	Min.	Avg.	Max.	Error Rate [%]
V_Lq_ [µV] (flow)	11.05	11.062	11.07	11.081	0.28	11.065	11.078	11.095	1.35
V_Lq_ [µV] (no flow)	0.000	0.12	0.12	0.153	1.38	0.140	0.150	0.180	1.63

Footnotes: min. (minimum), max (maximum), and avg (average).

**Table 2:** Comparison of capacitance-based and Lorentz-force-based sensing methods.

Category	Capacitive-Based Sensing	Lorentz Force-Based Sensing
detection target	presence of liquid	presence of liquid and flow rate/flow velocity
primary physical quantity	permittivity (ε)	charge transport (q), flow velocity (v), magnetic field (B)
effect of high-permittivity fluids	very significant	relatively small
flow rate/velocity detection	limited	direct detection possible
dependence on magnetic field	none	required
role in this study	auxiliary indicator for comparison and control group setup	core sensing mechanism

**Table 3:** Capacitance measurement results for liquid presence in the drainage tube, used for comparison with the sensor system.

Detection Signal	Calculation	Simulation Results				Measurement Results			
		Min.	Avg.	Max.	Error Rate [%]	Min.	Avg.	Max.	Error Rate [%]
C_Lq_ [pF] (flow)	19.1	19,312	19.14	19.16	0.31	19.18	19.22	19.26	3.30
C_Lq_ [pF] (no-flow)	0.00	0.30	0.50	0.60	3.14	0.40	0.55	0.80	4.20

Footnotes: min. (minimum), max (maximum), and avg (average).

**Table 4:** Comparison between previous studies and the proposed sensor system.

Ref [#]	Application Field	Sensor Method	Limitations	Distinction of This Study
[6]	IV line occlusion detection	pressure/flow sensor	wired connection, risk of infection	wireless, non-invasive approach
[7]	CSF drainage monitoring	pressure sensor	invasive, high infection risk	externally attachable sensor for improved safety
[8]	thoracic drainage monitoring	pressure-flow sensor	high cost, complex installation	low-cost, simple installation
[9]	smart drainage system	flow sensor + wired monitoring	no real-time remote monitoring	Bluetooth-based wireless transmission
[10]	post-craniotomy drainage management	capacitance sensor	single-parameter detection only, disposable design	this study employs Lorentz-force-based sensing + capacitance + potential difference + flow, velocity, and pressure monitoring, with reusable design
this works	paracentesis drainage monitoring	Lorentz force, capacitance, potential difference, flow, velocity, pressure sensors + wireless communication	currently tested only in simulated environment	non-invasive, wireless, multi-parameter monitoring, reusable, with real-time alarm system

## Data Availability

The original contributions presented in this study are included in the article/Supplementary Material. Further inquiries can be directed to the corresponding authors.

## Supplementary Materials

The following supporting information can be downloaded at: https://www.mdpi.com/article/10.3390/bios16020078/s1, Video S1.
